# The 2017 Network Tools and Applications in Biology (NETTAB) workshop: aims, topics and outcomes

**DOI:** 10.1186/s12859-019-2681-0

**Published:** 2019-04-18

**Authors:** Paolo Romano, Arnaud Céol, Andreas Dräger, Antonino Fiannaca, Rosalba Giugno, Massimo La Rosa, Luciano Milanesi, Ulrich Pfeffer, Riccardo Rizzo, Soo-Yong Shin, Junfeng Xia, Alfonso Urso

**Affiliations:** 1 IRCCS Ospedale Policlinico San Martino, Largo Rosanna Benzi 10, Genova, I-16132 Italy; 2 European Institute of Oncology IRCCS, Milan, 20141 Italy; 3 Computational Systems Biology of Infection and Antimicrobial-Resistant Pathogens, Center for Bioinformatics Tübingen (ZBIT), Tübingen, 72074 Germany; 4 Department of Computer Science, University of Tübingen, Tübingen, 72074 Germany; 5 ICAR-CNR, Institute for high performance computing and networking, National Research Council of Italy, Palermo, 90146 Italy; 6 Department of Computer Science, University of Verona, Verona, 37134 Italy; 7 ITB-CNR, Institute of biomedical technologies, National Research Council of Italy, Segrate (MI), 20090 Italy; 8 Department of Digital Health, SAIHST, Sungkyunkwan University, Seoul, 03063 South Korea; 9 Institutes of Physical Science and Information Technology, Anhui University, Hefei, 230601 China

## Abstract

The 17th International NETTAB workshop was held in Palermo, Italy, on October 16-18, 2017. The special topic for the meeting was “Methods, tools and platforms for Personalised Medicine in the Big Data Era”, but the traditional topics of the meeting series were also included in the event. About 40 scientific contributions were presented, including four keynote lectures, five guest lectures, and many oral communications and posters. Also, three tutorials were organised before and after the workshop. Full papers from some of the best works presented in Palermo were submitted for this Supplement of BMC Bioinformatics. Here, we provide an overview of meeting aims and scope. We also shortly introduce selected papers that have been accepted for publication in this Supplement, for a complete presentation of the outcomes of the meeting.

## NETTAB workshops

Network Tools and Applications in Biology (NETTAB) Workshops are a series of International meetings held annually in Italy since 2001 with the twofold aim of studying and analysing the impact of innovative Information and Communication Technologies (ICTs) on bioinformatics and biomedical research and of introducing them to researchers who did not yet acquire a sufficient expertise on them [1]. Scientific sessions are focused on tools, systems and applications that can be conceived and developed by adopting some given ICTs, as well as their expected impacts in Life Sciences. Sessions are usually introduced by keynote lectures and completed by selected oral communications. Discussions within oral and poster sessions allow participants to present and discuss their work and ideas on the main workshop topics. NETTAB workshops also includes some tutorials on cutting-edge technologies open to both young and senior researchers. Tutorial themes change every year in order to cope with on-going technological and scientific innovation in the field.

## NETTAB 2017

### Scope and rationale

The NETTAB 2017 workshop was held on October 16-18, 2017, in Palermo, Italy. Its main focus was on the study of methods, tools, and platforms for personalised medicine, in the context of big data. Personalised medicine aims at combining heterogeneous data collected from different sources, such as genetic heritage, lifestyle and environmental context, to advance disease understanding, diagnosis and treatment, and ensure delivery of appropriate therapies. In the last decade, it has been spent a big effort on collecting and exploiting the so called big data, extracted from clinical, imaging and “omics” data, as well as from electronic health records and mobile or wearable devices. Today, an open challenge in personalised medicine is going beyond limits given by some technical, ethical and political barriers, to ensure an efficient and reliable use of the big data in medical practice. Of course, development of new techniques for big data analysis and the introduction of platforms suitable for data integration, transformation and information sharing, represent the needed tools for defining personalised diagnostic, prognostic and therapeutics guidelines and models, that could be adopted to improve life-style, treatment and care of patients. The workshop gave to participants the opportunity of introducing and discussing new methods, theoretical approaches, algorithms, tools, and platforms, with specific reference to the special topics of the event as well as on many other bioinformatics topics, as from tradition of NETTAB previous events. For a full list of topics, see the meeting web site [2].

### Scientific programme

The programme included four keynote lectures, as well as five special guest lectures and 14 oral communications. Keynote lectures were given by Anita Grigoriadis, Winston Hide, Inna Kuperstein, and Alfonso Valencia with a clear emphasis on the main scientific topics of the meeting.

Anita Grigoriadis, currently at the Kings College, London, as lecturer in Cancer Bioinformatics, gave a talk on “Interoperability of clinical, pathological and omics data to execute personalised medicine”. Translational research has seen an increasing trend towards omics techniques, imaging approaches, in combination with clinical and pathological data. These multifactorial data, both large in sample size and heterogeneous in context, needs to be integrated in a standardised, cost-effective, secure manner so that it can be utilised and searched by researchers and clinicians. In her talk, she presented solutions to support data management, the integration of digital microscopy and pathology, and illustrated the utility of R-shiny to make high-throughput data searchable.

Winston Hide is Professor of Computational Biology at University of Sheffield, UK. His keynote talk was titled “Making genomics Come true: How can we achieve real acceleration of genomics into medicine?”. Genomic research is now rapidly moving from single human genomes to deca, centi and even milligenome projects. With more ways to compare gene variation against a background, comes new methods to select variants and genes for their potential in prediction and impact for a disease. Gene hunting is still very much a fashion and genes represent tempting targets for drug development. But the boundaries must be pushed to embrace the growing realisation that genes work in cohorts and it is the interaction of these cohorts that drive the disease phenotype. Identifying and targeting pathways and processes that drive disease is the new black. To action discovery, ways must be addressed in which to benchmark selection of disease genes, pathways and processes. In turn we need to develop more efficient (read less ineffective) ways to select therapeutics that are likely to be acceptable for real health interventions. The talk presented how the author’s group addresses these challenges through commoning for data sharing, provenance, reproducibility and workflows, benchmarks for assessment of approaches, standardisation for pathway activity, and integrative approaches to discovering the relationships between therapeutic target prioritisation, network topology, pathway interaction, genome variation, disease modelling and drug repurposing.

Inna Kuperstein is a scientist at Institut Curie in Paris, working on systems biology of cancer and other human diseases. Her keynote talk was titled “Atlas of Cancer Signaling Network and NaviCell: Systems Biology resources for studying cancer biology”. Studying reciprocal regulations between cancer-related pathways is essential for understanding signaling rewiring during cancer evolution and in response to treatments. With this aim the Atlas of Cancer Signaling Network (ACSN) has been constructed, a resource of cancer signaling maps and tools with interactive web-based environment for navigation, curation and data visualisation. The integrated NaviCell web-based tool box allows to import and visualize heterogeneous omics data on top of the ACSN maps and to perform functional analysis of the maps. The talk presented that the analysis of multi-omics data together with cell signaling information helps finding personalised treatments. Moreover, has been shown how epithelial to mesenchymal transition (EMT) signaling network from the ACSN collection has been used for finding metastasis inducers in colon cancer through network analysis. The structural analysis of EMT signaling network allowed highlighting the network organisation principles and complexity reduction up to core regulatory routes. Using the reduced network single and double mutants for achieving the metastasis phenotype have been modelled, and a prediction of a combination of p53 knock-out and overexpression of Notch would induce metastasis and suggested the molecular mechanism. This prediction lead to generation of colon cancer mice model with metastases in distant organs.

Alfonso Valencia is the Director of Life Sciences Department at the Barcelona Supercomputing Center. He is also the director of the Spanish National Bioinformatics Institute (INB-ISCIII). His keynote talk was titled “Disease Comorbidities and network based approaches”. The talk described new computational approaches to deal with inverse and direct cancer comorbidity in people with other complex diseases, as Alzheimer, Parkinson, autism, multiple sclerosis and type II diabetes.

The first guest lecture was given by Raffaele Giancarlo, Professor at the University of Palermo, Italy, who talked about “Getting Beyond Proof of Principle for Big Data Technologies in Bioinformatics: MapReduce Algorithmic, Programming and Architectural issues”. In his talk, he illustrated a scenario where a transition from the old High Performance Computing (HPC) in Bioinformatics paradigm to the new one of Cloud Computing, is achieved by using the MapReduce programming paradigm, which is in turn supported by Hadoop and Spark Middleware.

The second guest lecture was done by Francis Ouellette, Genome Quebec, Canada. In his talk, titled “Open Data is Essential for Personalised Medicine”, he gave an overview about the strategic issue of the Open Data for Personalised Medicine, with a particular focus on the use of Open Data in genomics, describing several scientific initiatives developed in Canada.

The third guest lecture lecture was given by Alexander Kel, GeneXplain, Germany. The talk, titled “Walking pathways in cancer”, discussed the evolutional advantages of the structural plasticity of gene regulatory networks as well as the high price such systems have to pay for this plasticity, for terrible diseases such as cancer. Often the non-reversible structural changes of the regulatory networks due to an epigenomic evolution of genome regulatory regions cause transformations in the system switching the normal state into a disease state. Such structural network changes are called walking pathways. The analysis of this phenomenon helps us to understand the mechanisms of molecular switches (e.g., between programs of cell death and programs of cell survival) and to identify prospective drug targets to treat cancer.

The fourth guest lecture, titled “Big data and cognitive computing in healthcare: weathering the perfect storm”, was done by Matthias Reumann, IBM Research, Zurich, Switzerland. The talk discussed, from an industrial perspective, the convergence of data analytics, sophisticated modelling approaches and cognitive computing to solve the big data challenges in healthcare and life sciences. In this context cognitive computing is a promising path to make the analytical results transparent and the power of big data can only be unleashed by embracing new approaches in data-driven analysis within a cognitive computing environment. This creates a holistic view that places big data analytics into the context of the accumulated knowledge of the scientific community.

The last guest lecture was given by Luana Licata, ELIXIR-IIB, Italy. The lecture, titled “ELIXIR-IIB: the Italian Infrastructure for Bioinformatics: a growing support to national and international research in life sciences”, gave an overview on the ELIXIR Italian node and infrastructure for bioinformatics. The node is coordinated by the National Research Council and currently includes 17 centers of excellence among which are research institutes, universities and technological institutions. The infrastructure supports the exchange and development of skills, and the integration of publicly available and internationally recognised Italian bioinformatics resources within the European infrastructure.

### Tutorials

Three tutorials were held before and after the workshop. Two of them were held in parallel on October 16 morning.

The first tutorial was offered by INdAM GNCS Project: “Efficient Algorithms and Techniques for the Organization, Management and Analysis of Biological Big Data”. The tutorial, titled “An introductory tour on Big Data, Big Data technologies, and Big Data applications in Biology and Medicine”, was given by Simona Rombo, University of Palermo, Italy. The tutorial aimed at providing an overview of the Big Data technologies, by specifying some technical aspects and by illustrating how these technologies may be applied in the bioinformatics/biomedical context. It first focused on introducing the audience to the notion of Big Data. Then the Map-Reduce paradigm was explained, by also discussing how it has been implemented on the Apache Hadoop and Spark frameworks. Moreover, the two frameworks were compared in terms of their advantages and drawbacks, by explaining when each of them is more suitable to be applied than the other one. The second part of the tutorial, explained why the relational model cannot be applied for database design in the Big Data era, and the notion of NoSQL databases was illustrated as well as some of the most famous examples, such as Cassandra, MongoDB, HBase, etc.. Finally, some applications of Big Data technologies in the context of Biology and Medicine was shown.

The second tutorial was offered by the CNR InterOmics Flagship project. The tutorial, titled “Biological network-based analysis of omics for precision medicine: overview, interaction databases and network diffusion approaches”, was given by Ettore Mosca, Institute of Biomedical Technologies, National Research Council of Italy, Milan, Italy. The tutorial illustrated how the known molecular interactions can be useful in the interpretation of “omics” data for precision medicine, with a main focus on data referable to genes. The first part introduced broad objectives and principles of biological network-based analyses of “omics” data. Then, an overview of the state-of-the art of current datasets from which networks can be defined was given, also in term of their completeness in relation to the coverage of high-throughput technologies and the issues in mapping “omics” data to biological networks. The second part focused on network diffusion-based approaches and illustrated how the principles behind this class of methods have been recently applied to several problems, including patient stratification and gene module extraction. Throughout the tutorial, benefits, limitations and open issues in the field of network-based analysis were underlined, including also software availability and computational cost.

The last tutorial was offered by ELIXIR-IIB. The tutorial, titled “Biological Networks: data analysis, visualisation and medical application”, was given by Inna Kuperstein, Institute Curie, Paris, France, Alberto Calderone and Luana Licata, ELIXIR-IIB, Italy. The goal of this tutorial was to provide an overview on network construction, data analysis and modelling in bio-medical research. The participants were introduced to molecular and causal interactions field, how these data are curated and which are the international standards and the ontologies adopted to describe these data. A large number of resources and methodologies were introduced. Hands-on sessions were provided where participants were asked to analyse a dataset in the context of network analysis and modelling. Moreover, available methods and resources for integrated analysis and visualisation of different multi-omics data were also shown. Participants developed an understanding not only on static networks but also on discrete and continuous dynamical networks. They also understood how to simulate basic systems in order to derive meaningful information related to cancer.

## Introduction to selected papers

### Selection of best papers

Fifteen full papers were selected for publication in this Supplement after the workshop. The Guest Editors of the Supplement, Paolo Romano and Alfonso Urso, selected an Editorial Board by paying attention that topics of submitted manuscripts were properly covered.The Editorial Board included the following Associated Editors:

- Ludmil Alexandrov, University of California San Diego, USA
- Arnaud Céol, European Institute of Oncology IRCCS, Milan, Italy
- Andreas Dräger, Center for Bioinformatics Tübingen (ZBIT) and University of Tübingen, Germany
- Antonino Fiannaca, ICAR-CNR, Palermo, Italy
- Rosalba Giugno, University of Verona, Italy
- Massimo La Rosa, ICAR-CNR, Palermo, Italy
- Frédérique Lisacek, Swiss Institute of Bioinformatics, Switzerland
- Donato Malerba, University of Bari “Aldo Moro”, Italy
- Luciano Milanesi, ITB-CNR, Segrate (MI), Italy
- Riccardo Rizzo, ICAR-CNR, Palermo, Italy
- Paolo Romano, IRCCS Ospedale Policlinico San Martino, Genoa, Italy
- Soo-Yong Shin, Sungkyunkwan University, South Korea
- Andrea Splendiani, Novartis, Basel, Switzerland
- Alfonso Urso, ICAR-CNR, Palermo, Italy
- Wen Zhang, Icahn School of Medicine at Mount Sinai, New York, USA
- Junfeng Xia, Anhui University, China.

Associated Editors managed the reviewing process for one manuscript, which was assigned to one of them according to his/her expertise. Two or more referees were selected for each submission: 49 referees were involved overall. Authors were invited to submit an improved version of their manuscript after the reviews were collected. Associated Editors made the final recommendation for each manuscript and, at the end of this process, fifteen papers out of the eighteen submitted were accepted and included in this Supplement.

### Brief description of selected best papers

Network maps are *de facto* tools to understand the cell organisation [3, 4]. But the study of individual networks is yet limited. Supporting evidence for the effect of alterations and perturbations of the system can be revealed by looking at the interplay between different kinds of networks. To illustrate this, Sompairac et al. [5] have integrated three maps (two signalling and one metabolic): the Atlas of Cancer Signalling Network [6] and ReconMap [7], a human metabolic network. Some findings were expected, like the presence of modules related to mitochondria metabolism and apoptosis, but other ones suggest novel insights on cancer progression, such as, among others, the identification of modules related to metabolism and invasion progress, pointing to an active role of the metabolism in the migration of cancer cells. In this article, what may surprise the reader is how much work is still required to make two maps talk together. In the last decades, the management of biological data has been leveraged by the development of numerous standards for data representation and exchange [8], and their adoption by versatile tools (see for instance [9]). More recently, the FAIR principles have been proposed, which promotes the Interoperability and the Reusability of data [10]. Despite all those efforts, the authors still needed to manage several programs, map identifiers from different source databases, and extract and convert numerous file formats. Thankfully, the resulting ACSN-ReconMap 2.0 map (which integrates signaling and metabolic networks) has been released and can be interrogated on the NaviCell platform [11], a web application that allows to explore large biological networks. The maps in NaviCell are static, therefore regions can be graphically isolated and easily visualised, to delimit for instance signaling pathways. The signaling and the metabolic networks from the ACSN-ReconMap are displayed on two different maps, but the authors made it easy to navigate from one to the other. This elegant implementation relies on the usage of “Google maps” features to identify one or more proteins on the map, zoom in or out, and offer links to the other map if the protein is available in both. This will be a valuable tool to identify disease related alterations that perturb the signaling metabolism cross-talk.

The paper “RIP-Chip analysis supports different roles for AGO2 and GW182 proteins in recruiting and processing microRNA targets” [12], by Perconti et al., analyses the role of the AGO2 and GW182 RISC proteins in miRNA regulatory activity on the MCF-7 human breast cancer cell line, combining the result of immunoprecipitation experiments, miRNA-target prediction databases and a machine learning algorithm. Authors suggest different features to distinguish between enriched and underrepresented genes in miRNA machinery when AGO2 or GW182 are taken into consideration. Furthermore, they point out that shorter mRNAs are most likely processed in ways that lead to degradation, whereas longer mRNAs tend to be stored in GW/P bodies.

Next Generation Sequencing techniques (NGS) strongly impacted biological studies and are widely used in life sciences. However, NGS results can be affected by many artifacts which can impact the quality of the input data and compromise the downstream analysis. These artifacts can have a wide range of causes, and one of them is contamination from known or unknown species, other than the sequencing target [13]. Sangiovanni et al. [14] started to address this problem by developing the first version of DecontaMiner tool in 2016; the tool allows the researchers to identify and study the sources of contamination. DecontaMiner can also be used as a filtering tool to remove low quality and non-human reads. In this new study, the authors started with a background analysis focused on the upstream and downstream contamination organisms, as reported in [15–17]. They also deeply analysed the other available tools for the detection of pathogens in sequencing data and studied their integration in NGS pipeline. From this analysis, the authors developed the idea of a tool that can combine the advantage of a simple integration with the processing pipeline, the configuration flexibility, and the effectiveness of the filtering action. For this reason, the original tool was upgraded to allow the user to run all the steps of the processing pipeline separately, and this also allows to fine tune all the processing parameters. Moreover, the authors conducted an in-depth analysis of the performances of DecontaMiner by comparing it with two available similar systems, namely TruePure and FastQScreen, showing its effectiveness regarding precision and recall, and highlighting the advantage of the proposed system.

Multiple studies have suggested obesity as a potential risk factor for postmenopausal breast cancer [18–21]. Driven by the aim to elucidate this association, Granata et al. [22] developed a method to identify significant deviations of critical metabolic reactions between lean and obese breast cancer patients using a systems biology approach. To this end, the authors integrate “transcriptomic data in a genome-scale metabolic model” of human adipocytes. This technique is used to analyse gene expression data in the context of a metabolic network, aiming to investigate the genetic causes of the observed metabolic phenotype. First, the authors gathered all necessary information from literature: a transcriptomic data set for the two subject groups, obese and lean postmenopausal Luminal-A breast cancer patients, and a cell-type specific model iAdipocytes1809 [23] of human adipocytes. Next, the fundamental idea of the work was to apply the algorithm by Lee et al. [24] to generate testable hypotheses by simulating the obtained constraint-based model. In contrast to so-called biased simulation methods, Lee’s algorithm has the advantage not to require any specific target function to maximise the “correlation between experimentally measured absolute gene expression data and predicted internal reaction fluxes” [24]. With their implementation of Lee’s approach, the authors were able to compare the model output to their gathered gene expression values using COBRA Toolbox 3.0 [25]. A comparison of the results of regular flux balance analysis to Lee’s method and experimental data (the production rates of non-esterified fatty acids) confirmed the reliability of their approach and led to the identification of five differentially expressed genes showing a significant association with specific functions in the breast cancer phenotype. By dividing each of their cohort of obese and lean patients into two subgroups before and after ingestion and calculating singular flux distributions, the authors could identify remarkable changes of flux directionality in solute transport reactions. The Cytoscape [26] app DyNet [27], made it then possible to visually analyse significantly changed activity patterns of the network topology for all four patient groups. The results showed that both lean networks possess higher reaction counts and higher connectivity of their nodes but with higher diversity compared to the obese versions. DyNet uses a rewiring metric, called Dn-score, to rank the variability of connections between metabolites via reactions. This Dn-score ranking identified that amino acid transport reactions are most different between the two patient groups. However, when applying the ranking on genes instead of reactions, the top 50 genes were annotated with functions in metabolism of fatty acid, amino acid, glutathione, and oxidative stress. Summarising, Granata et al. demonstrate how the combination of an unbiased algorithm for simulating genome-scale metabolic networks and a large variety of analysis methods can be used to determine the metabolic effect of obesity on the formation of Luminal-A breast cancer. Their work demonstrates a method to characterise Next Generation Sequencing data in a systemic context. This work could be beneficial for interpreting gene expression states in conjunction with metabolic states.

In the paper “Greedy de novo motif discovery to construct motif repositories for bacterial proteomes” [28], Khakzad et al., identified motifs in bacterial protein families through a greedy *de novo* approach combining available tools from the MEME suite [29]. They show the utility of such approach constructing a proteome-wide motif repository for Group A streptococcus (GAS) opening to the applicability of the method to all major human pathogens. They hypothesised that the method can identify functionally essential motifs involved in protein interactions. They highlighted results by working on proteins subjected to the host’s immune system which are under a strong evolutionary pressure. As proof of concept they show that M protein, which binds several plasma proteins, has indeed a motif with different conformations in several GAS strains.

The paper titled “Walking pathways with positive feedback loops reveal DNA methylation biomarkers of colorectal cancer” [30], by Kel et al., proposes a new bioinformatic method based on the modification of the master-regulator search algorithm [31] to search for “Walking Pathways”, i.e. for potential rewiring mechanisms in cancer pathways due to dynamic changes in the DNA methylation status of important gene regulatory regions. This methodology has been successfully used to find potential causal relationships between epigenetic changes (DNA methylations) in gene regulatory regions during cancer development that affect transcription factor binding sites (TFBS) and gene expression changes. The authors apply this methodology for bio-marker discovery in patients with colorectal cancer. This work performs a detailed multi-omics analysis of large volumes of RNA-seq and DNA methylation data, primarily generated by the SysCol project and published in previous publication [32].

If cancer is mainly due to bad luck [33] its probability is certainly influenced by external conditions such as exposure to carcinogens, life style and diet, factors already well known for example for breast cancer [34]. Etiological factors like the ultra violet component of sunlight for melanoma [35] contribute to cancer risk and leave a characteristic foot print in the tumor cell DNA by provoking specific mutational patterns. It has been known for a long time that *C*→*T* transitions are induced by UV-light in melanoma, prevalently at dipyrimidine sites (mostly CC) but only recently, the analysis of mutation patterns has been extended to all cancers. Two methods to classify mutation patterns have been developed considering either 96 [36] or 14 [37] combinations. The frequency of the single mutational contexts can be combined into a “signature” that is characteristic for different types of cancers and likely linked to etiological factors like UV-irradiation, cigarette smoke or age-related deamination of 5-methylcytosine. And this is exactly why we should care for mutational signatures: they can help to link etiological factors to orphan signatures and to identify external and endogenous factors that contribute to the development of specific cancers, a knowledge that could help to design prevention strategies. In addition, subclassifications of tumors according to their mutational signatures should be possible. In times of massive parallel sequencing, the analysis of mutational signatures will yield more and more insight by adding ever more tumor exome or whole genome sequences. Therefore the contribution of Krüger and Piro [38], titled “decompTumor2Sig: Identification of mutational signatures active in individual tumors”, who report on a simplified bioinformatic procedure to analyse mutational signatures is highly welcome, also because it considers the two approaches developed by Alexandrov et al. [36] and Shirashi et al. [37], the latter of which has so far obtained less attention.

Comparing RNA secondary structures is currently only possible for structures that do not include pseudoknots. In their paper “An algebraic language for RNA pseudoknots comparison”[39], Quadrini et al. introduce an unambiguous algebraic language for the representation of RNA secondary structures with arbitrary pseudoknots. The language grammar includes special operators for concatenation, nesting and crossing. By leveraging from this language, any RNA structure can be represented by a unique tree that can then be compared with any other RNA tree by means of a purpose similarity measure; this can be derived from existing methods for tree alignment. The actual implementation of comparison algorithms and tools is only planned at the moment.

Gilbert et al. [40], in the paper “Spatial quorum sensing modelling using coloured hybrid Petri nets and simulative model checking”, introduce a computational models for simulating the quoring sensing mechanism responsible for the production of biofilm in bacteria. Quoring sensing is a communication mechanism based on a process of stimulus and response correlated to population density of bacteria. This process, activated by a signalling molecule called autoinducer, causes, among the others effects, the production of biofilm, which, in turn, can act as a physical protection against antibiotics. The proposed model, based on coloured Petri nets, has been developed and validated, and the results confirm the expected behaviour, that is biofilm formation is related to bacterial density, increasing its volume in high populated areas.

Haplotypes have a crucial role in fully characterizing the genome of individuals for disease diagnosis or association studies. However, constructing full diploid sequences is known as NP-hard problem [41]. Lots of approaches such as rule-based approach, expectation-maximisation, Bayesian methods, hidden Markov models, and evolutionary computation, have been proposed to construct diploid sequences (known as haplotype assembly) [42]. The paper “GenHap: A NovelComputational Method Based on Genetic Algorithms for Haplotype Assembly” [43], by Tangherloni et al., implements GenHap based on genetic algorithms which have proven to effectively search practical solution for NP-hard problem. They formulated haplotype assembly as a weighted minimum correction problem. Though the weak point of genetic algorithm approach is that they require a lot of computational time, the proposed GenHap demonstrated shorter computational times compared to the previous method.

Amyotrophic lateral sclerosis (ALS) is a severe neurodegenerative disease with a life expectancy from three to five years. Diagnosis and prognosis for individual ALS patients is challenging due to the extreme variability of symptoms and impairment levels. As a consequence, stratification of patients into meaningful subgroups is also difficult. In their work “A Dynamic Bayesian Network model for the simulation of Amyotrophic Lateral Sclerosis progression”[44], Zandonà et al. demonstrate the potential of Dynamic Bayesian Networks (DBNs) in this context. Starting from the Pooled Resource Open-Access ALS Clinical Trials Database (PRO-ACT)[45], they developed a DBN model including 4,500 ALS patients. The model was used to calculate probabilistic relationships among clinical variables, to identify risk factors and to simulate the evolution of an ALS cohort and predict survival and time to impairment of vital functions. A first attempt to stratify patients by risk factors and simulate the progression of ALS subgroups was also carried out. The authors demonstrate that the adoption of DBNs can indeed allow the analysis of relationships among clinical variables, support the stratification of patients and lead to the achievement of relevant new insights.

One the aspects that are relevant to personalised medicine is the drug response variability among individuals. Such variability is influenced by a variety of factors (e.g.: age, nutrition, health status, environmental factors) but a key role is played by genetics. Pharmacogenomics builds on the comprehensiveness of genomics measurements to identify relations between genetic variations and drug response [46, 47]. Pharmacogenomics information is typically made available in the scientific literature and is in turn curated and organized in specialised databases (e.g.: Pharmagkb [48]). As we move toward more personalised and data-centric approaches in medicine, there is a rising need to integrate such information, across multiple sources, in an agile way. Evidence or putative evidence of pharmacogenomics interactions may in fact come from direct Electronic Health Record (EHR) analysis. Given the increasing availability of genomic testing and patient-centric health platforms, even more sources of such information may arise. Monnin et al. present “PGxO and PGxLOD: a reconciliation of pharmacogenomic knowledge of various provenances, enabling further comparison”[49], which addresses such need by providing an infrastructure, based on semantic web technologies, where different pharmacogenomics “facts” can be combined from different sources. In particular PGxO keeps tracks of provenance information, and provides a unified conceptual framework, as well as mapping rules, to integrate information from databases, literature and EHRs. The paper reports both results and current challenges and limitations in performing such integration.

Ferraro Petrillo et al., in their paper “Analyzing big datasets of genomic sequences: fast and scalable collection of k-mer statistics” [50], present “FastKmer” an algorithm that deals with the extraction of k-mers, with arbitrary value of k, from biological sequences, both genomics and metagenomics. FastKmer is implemented to run with the Spark framework, following a distributed approach for kmer counting based on the MapReduce paradigm. Authors, by means of extensive tests, stated FastKmer is the fastest k-mer statistic distributed algorithm so far. In the near future, moreover, authors plan to use the internal architecture of FastKmer for other bioinformatics applications, such as distributed alignment-free algorithms

Navas-Delgado et al., in their paper “VIGLA-M: Visual gene expression data analytics” [51], introduce a promising tool termed VIGLA-M for use in clinical assays to enable physicians the exploration of gene expression data in patients. The authors test VIGLA-M in the context of a clinical assay with metastatic melanoma patients. To support large scale clinical assays, a parallel version of the tool is also developed with Apache Spark. Although this tool is tested on a small set of melanoma patients, it is intended to support similar cases in other diseases where gene expression analysis would help in discovering new biomarkers.

Fassetti et al., in their paper “FEDRO: a software tool for the automatic discovery of candidate ORFs in plants with c →u RNA editing” [52], present FEDRO, a software tool written in Java able to predict RNA editing events for gene expression in plants organelles. RNA editing alters the direct transfer of genetic information from DNA to proteins in plants organelles, due to the introduction of differences between RNAs and the corresponding DNA coding sequences. Existing software tools, which are successful for the search of genes in other organisms, not always can correctly perform this task in plants organellar genomes. FEDRO implements a novel strategy to generate candidate Open Reading Frames (ORFs), based on the simulation of *c*→*u* RNA editing in the mitochondria of plants. The end result is the prediction of proteins which have not been discovered and annotated yet [53]. Authors apply FEDRO on the mtDNA of *Oryza sativa*, suggesting a set of 45 novel putative ORFs to be verified by experts.

## Abbreviations

ACSN: Atlas of cancer signaling network
ALS: Amyotrophic lateral sclerosis
CNR: Consiglio nazionale delle ricerche
DBN: Dynamic Bayesian network
EHR: Electronic health record
FAIR: Findable, accessible, interoperable, reusable
GAS: Group A streptococcus
HPC: High performance computing
ICT: Information and communication technology
INB-ISCIII: Spanish national bioinformatics institute
NETTAB: Network tools and applications in biology
NGS: Next generation sequencing
ORF: Open reading frame
PGxO: Pharmacogenomics ontology
PRO-ACT: Pooled resource open-access ALS clinical trials database
TFBS: Transcription factor binding site
